# Maxillomandibular giant osteosclerotic lesions

**DOI:** 10.1590/1678-7757-2017-0535

**Published:** 2018-05-29

**Authors:** Constantino LEDESMA-MONTES, María Dolores JIMÉNEZ-FARFÁN, Juan Carlos HERNÁNDEZ-GUERRERO

**Affiliations:** 1 Universidad Nacional Autónoma de México, Facultad de Odontología, Ciudad del México, México.

**Keywords:** Bone density, Osteosclerosis, Osteitis, Developmental bone diseases

## Abstract

Giant Osteosclerotic Lesions (GOLs) are a group of rarely reported intraosseous lesions. Their precise diagnosis is important since they can be confused with malignant neoplasms.

**Objective:**

This retrospective study aimed to record and analyze the clinical and radiographic Giant Osteosclerotic Lesions (GOLs) detected in the maxillomandibular area of patients attending to our institution. Materials and Methods: Informed consent from the patients was obtained and those cases of 2.5 cm or larger lesions with radiopaque or mixed (radiolucid-radiopaque) appearance located in the maxillofacial bones were selected. Assessed parameters were: age, gender, radiographic aspect, shape, borders, size, location and relations to roots. Lesions were classified as radicular, apical, interradicular, interradicular-apical, radicular-apical or located in a previous teeth extraction area. Additionally, several osseous and dental developmental alterations (DDAs) were assessed.

**Results:**

Seventeen radiopacities in 14 patients were found and were located almost exclusively in mandible and were two types: idiopathic osteosclerosis and condensing osteitis. GOLs were more frequent in females, and in the anterior and premolar zones. 94.2% of GOLs were qualified as idiopathic osteosclerosis and one case was condensing osteitis. All studied cases showed different osseous and dental developmental alterations (DDAs). The most common were: Microdontia, hypodontia, pulp stones, macrodontia and variations in the mental foramina.

**Conclusions:**

GOLs must be differentiated from other radiopaque benign and malignant tumors. Condensing osteitis, was considered an anomalous osseous response induced by a chronic low-grade inflammatory stimulus. For development of idiopathic osteosclerosis, two possible mechanisms could be related. The first is modification of the normal turnover with excessive osseous deposition. The second mechanism will prevent the normal bone resorption, arresting the osseous breakdown process.

## Introduction

During many years, two radiopaque entities were confused and known with different names: osteosclerosis, sclerosing osteitis, condensing osteitis, bone whorls, bone eburnation, chronic focal sclerosing osteomyelitis and idiopathic osteosclerosis.^25^ In 1985, Stafne divided these lesions in two entities: Condensing osteitis (CO) and osteosclerosis.^12^ CO lesions were known since the Brower, et al.^7^ (1974) report. They presented the clinical, roentgenologic and microscopic findings of radiopaque, fusiform, painful, slow growing, clavicular swellings in two patients and, later, more cases were added^8^. COs are intrabony, radiopaque lesions associated to inflammatory processes, more commonly to low-grade inflammation and, to date, etiological factors associated to development of COs are numerous.^1^ ^,^ ^3^ ^,^ ^5^ ^,^ ^7^ ^,^ ^8^ ^,^ ^20^ ^,^ ^26^ COs are infrequent lesions in the general population, they are more common in females and most of them appear as radiographic images smaller than 2 cm.^5^ ^,^ ^20^ ^,^ ^26^ They are usually found in mandible and posterior regions are more commonly involved.^5^ ^,^ ^7^ ^,^ ^20^ ^,^ ^26^ ^,^ ^27^

Idiopathic osteosclerosis (IO) lesions are reported in pelvis, long bones, maxilla and mandible mainly as asymptomatic, non-expansible, radiopaque or mixed images diagnosed at any age. IO was found in both genders and lacks any relationship to inflammatory, infectious or traumatic stimulus. It is more prevalent in females, more frequently found in mandible and posterior areas and it is considered as an anatomic variant or a developmental osseous entity.^9^ ^-^ ^11^ ^,^ ^18^ ^,^ ^19^ ^,^ ^23^

In 1973, Smith^24^ reported two cases of large osteosclerotic lesions located in the acetabular area and ileum suggesting, the term “giant bone island” to those radiopacities measuring 2 cm. Since then, numerous cases were reported in femur, tibia, ribs, pelvis, spine, sacrum, ilium and iliac bones.^4^ ^,^ ^6^ ^,^ ^21^ ^,^ ^22^ ^,^ ^24^ These lesions measured from 2 to 10.5 cm^21^ and were more frequently found in male patients.^6^ To date, reports on the incidence in the general population are not published.

Kawai, et al.^17^ (1996) wrote on the features of 21 intraosseous lesions larger than 2 cm, located in the maxillomandibular area and described their clinical, radiographic and microscopic features. This is the sole report on these lesions found in the maxillomandibular area.

During the review of the radiographs from the patients seeking stomatological attention to the Oral Diagnosis Clinic of the *División de Estudios de Posgrado e Investigación* in our institution, we observed that large intraosseous osteosclerotic lesions were present in some patients. For this reason, the aims of this study were to record and to analyze the clinico-radiographic features detected in all 2.5 cm or larger radiopaque or mixed (radiolucid-radiopaque) images detected in the maxillomandibular zone of the patients, hypothesizing that these alterations could be of clinical, pathological and academic interest.

## Material and methods

This study included all the patients who sought stomatological attention during one year in the Admission and Diagnosis Clinic of our institution. A panoramic radiograph was done, and all of them, or their parents, signed a Letter of Consent giving permission to use their data and images for research purposes only. Additionally, the Ethics Committee approved the research protocol (CIE/02/10/06/2016/05).

All cases were of radiopaque or mixed appearance measuring 2.5 cm or more were analyzed and those diagnosed as Giant Osteosclerotic Lesions (GOLs) were included. Applying the mentioned features, some of the selected lesions were diagnosed as IO and other with clinico-radiographic features of CO were also incorporated. For diagnosis of IO, the parameters of the MacDonald-Yankowski study^19^ were applied. A caliper was used for measurement of the micro and macrodontic teeth.

As COs, we diagnosed all radiopaque or mixed intraosseous images associated to teeth with deep caries or large restorations, lesions located in edentulous regions related to dental extraction and those located around teeth showing marked malposition or served as abutments for fixed bridges or partial dentures. Also, images related to teeth under orthodontic forces or those associated to resorption of the adjacent teeth were also included.

Assessed parameters of the GOL studied were gender and age of the patients. Other parameters were: side, radiopaque or mixed appearance, shape, homogeneous or heterogeneous core, borders, size, location and relation to roots or bone. Classification: Analyzed lesions were grouped as radicular, apical, interradicular, interradicular and apical, associated to endodontic treatment and located in a dental extraction area.

Data recorded from affected patients were analyzed with the SPSS program (22.0 v) and p<0.05 was considered significant.

## Results

**Figure 1:**
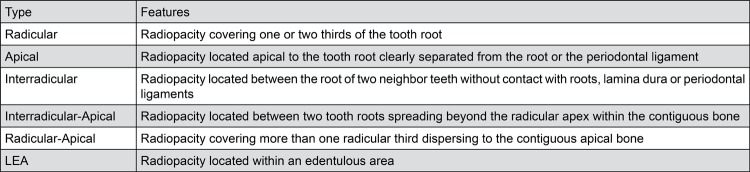
Radiographic types of GOLs

From the 6,340 reviewed panoramic radiographs, 17 images were compatible with GOLs, and 14 patients were diagnosed. None of the patients presented clinical or roentgenologic features of the Gardner’s syndrome or were affected by any bone disorder. Figure 1 shows the different types of GOLs according to their relationship to adjacent tooth or teeth, and in Table 1 the main demographic data is presented.

**Table 1:** Demographics of the analyzed GOL lesions

Diagnosis	17 IO (64.7%); 6 OCs (35.3%)	
Gender:	14 females= 64.3% 5 males= 35.7%	Age:12-67 years mean= 32.5 years; SD±15.8 years
Size:	2.75-8.25 cm mean= 4.74 cm SD±0.84 cm	30 years or younger 4.82 cm Older 4.80 cm
Location:	Maxilla (n=1; 5.9%)	Anterior (n=6; 35.3%).
	Mandible (n=16; 94.1%).	Premolar-molar (n=4; 23.5%). Premolar, anterior-premolar and anterior-molar zones (n=2 cases each; 11.7% respectively). Molar area (n=1; 5.8%).
	Apical and apical-interradicular (n=4 each; 23.5% respectively), radicular-interradicular (n=2; XX%), radicular (n=2; X%), in edentulous areas (n=5; X%)	
Radiographic Features:	Density: 7 radiopaque (41.2%), 10 mixed (58.8%),	Borders: Distinct (n=13; 76.5%), Ill-defined (n=2; 23.5%).
Shape:	Irregular (n=9; 52.9%), Oval (n=4; 23.5%), round (n=3; 17.6%), Triangular (n=1; 5.9%).	Homogeneity: Homogeneous (n=1; 5.9%) Heterogeneous (n=16; 94.1%)
Associations to:	Periodontal ligament (n=16; 94.1%), healthy teeth (n=10; 58.8%), in place of a congenitally missing tooth, surrounding an impacted tooth and endodontic treatment (1 case each; 5.9% respectively)	

RO= Radiopaque, MIX = Mixed, WDL= Well-defined limits, IDL= Ill-defined limits, NH= Non-homogeneous,

Rou= Round, Tri= Triangular. Irr= Irregular; LEA= Located within an edentulous area

Comparing mean size of the lesions in 30 year-old or younger patients and their size in older people, there were no statistically significance (p>0.05). Comparing the frequency of IO and CO, statistical difference was found (p<0.05). Also, frequency of GOLs in maxilla and mandible was statistically different (p<0.001).

All GOLs were considered as incidental radiographic findings, since they were all asymptomatic at first appointment and patients were unaware about their presence or development. Additionally, at clinical review, palpation of the involved zones showed no bony expansion, and even those cases located in a dental extraction area were painless lesions at exploration or interrogation.

**Figure 2:**
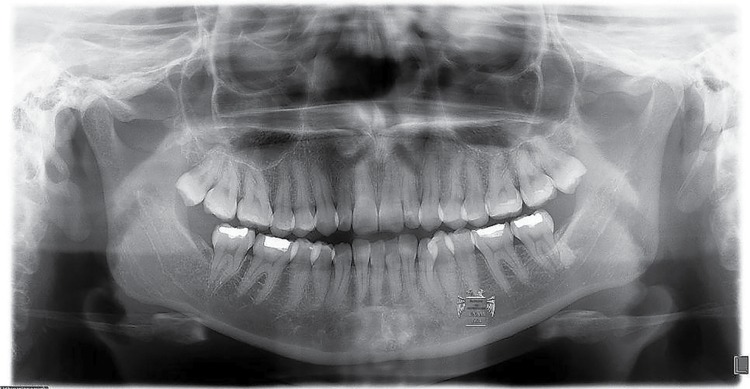
Case 6. Radiopaque gigantic lesion located in the anterior mandible. Two microdontic lateral incisors, dilaceration of the right canine and a small radiopaque zone in the mandibular right third molar correspondent to a condensing osteitis are seen

**Figure 3:**
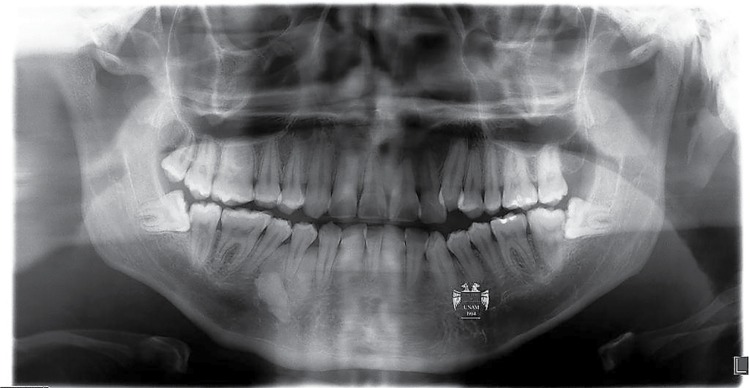
Case 2. Radiopaque giant lesion associated to a supernumerary right mandibular premolar. Two mandibular molars, two maxillary first molars and both maxillary lateral incisors are macrodonts. Additionally, left maxillary canine and both premolars are microdontic, including right mandibular second molar

**Figure 4:**
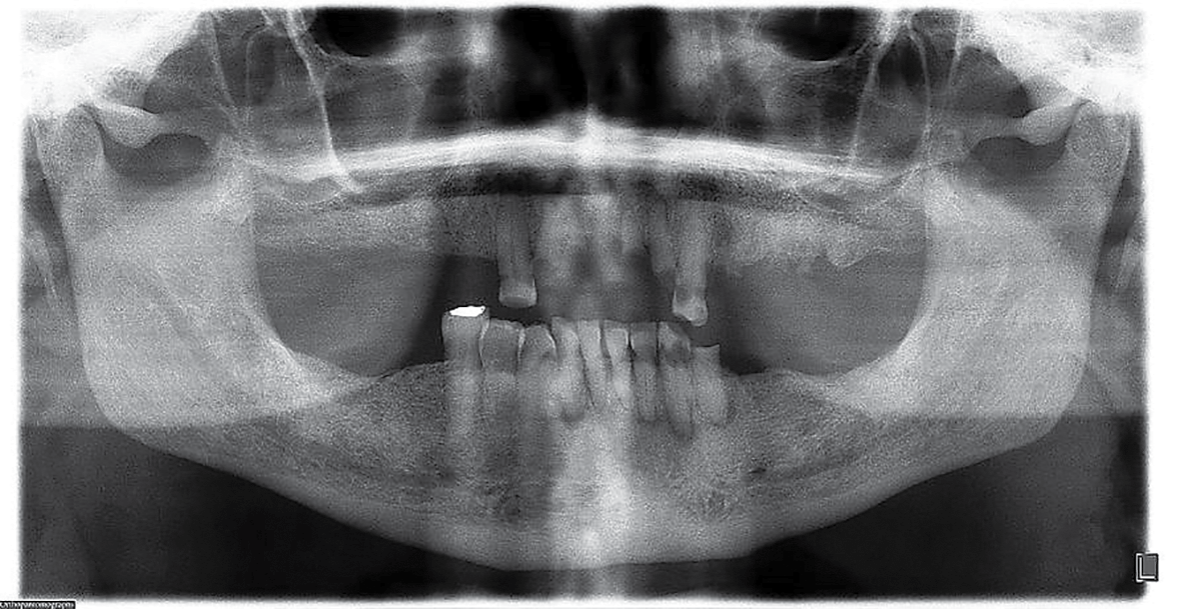
Case 14. Both mandibular premolar-molar zones are occupied by two giant mixed images associated to dental extraction. Maxillary left lateral incisor and mandibular left canine were congenitally missing

**Figure 5:**
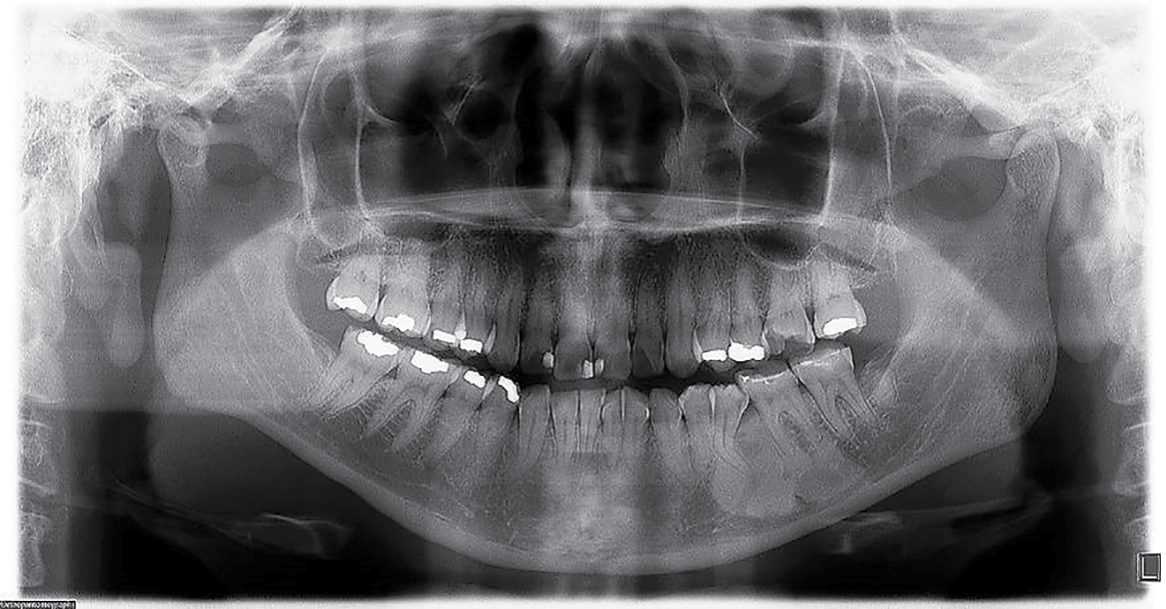
Case 11. The giant osteosclerotic lesion is located around the root of the maxillary right central incisor. Additionally, right lateral incisor and mandibular left canine are hypodontic. Note an enamel pearl in the neck of the mandibular right first molar

**Figure 6:**
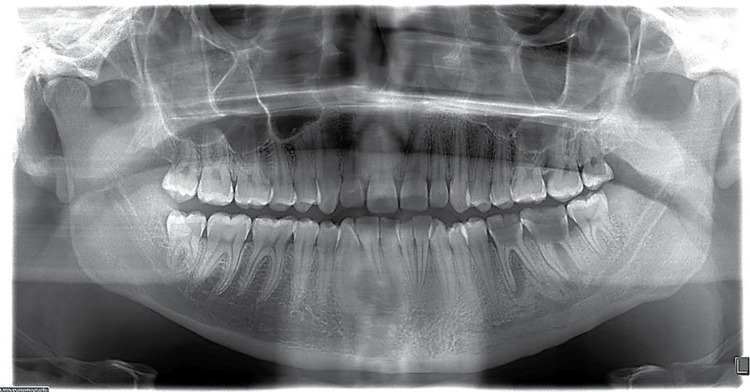
Case 5. A large radiopaque well-defined image is seen in relation with the dilacerated mandibular left second premolar and first molar. A microdontic maxillary left canine is also observed. The small radiopaque mass in the mandibular left third molar area corresponded to a condensing osteitis lesion

Radiodensity of the images was statistically significant (p=0.01). Figure 2 shows a lesion in an apical location; one of the four cases seen in apical-interradicular position is in Figure 3; and Figure 4 illustrates an image in radicular-interradicular place. Radicular position is shown in Figure 5, and a CO located in an edentulous area is in Figure 6.

**Figure 7:**
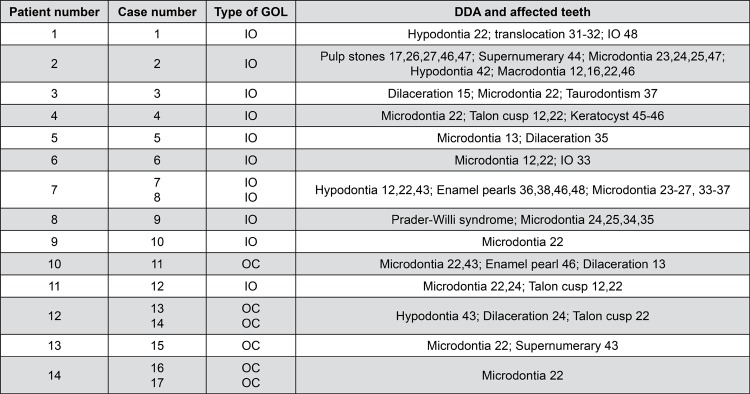
Patients with GOLs and dental developmental alterations

Detailed data on the type of GOL and the type of DDA found in each of the studied cases are presented in Figure 7.

**Figure 8:**
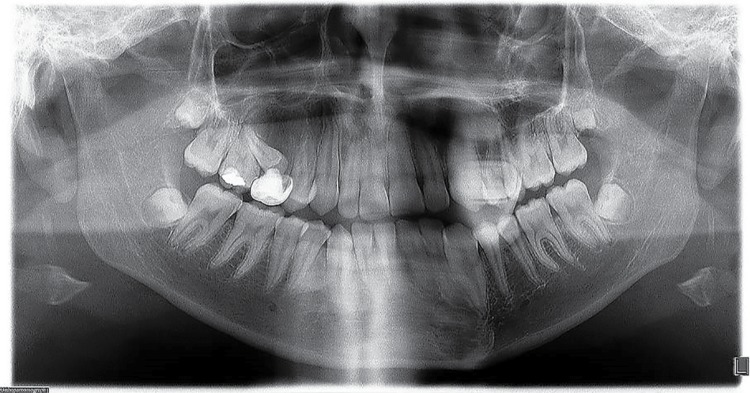
Case 8. This radiograph corresponds to a female patient previously diagnosed with Prader-Willi syndrome. A mixed image is observed in the left mandibular region, generating displacement of the right mandibular incisors and canine and both premolars. Also, the left maxillary second premolar is displaced and unerupted. Maxillary and mandibular left premolars are microdontic

**Figure 9:**
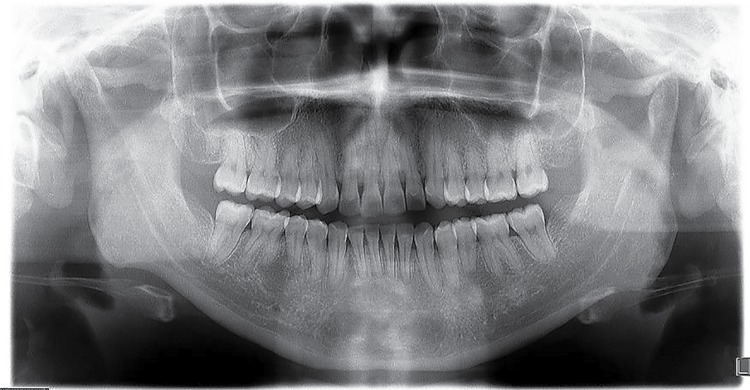
Case 4. A mixed, intraosseous, well-defined image from right mandibular canine to its contralateral counterpart is seen. A mixed, well-defined lesion is seen in the mandibular right premolar-molar zone. This image is the cicatrization area corresponding to a lesion previously diagnosed as an odontogenic keratocyst

Interestingly, we detected several cases showing unusual alterations in the mandibular foramina. One left supernumerary premolar in case 2 was detected, with no radiographic evidence of both foramina below the roots of the normal appearing premolars, and after careful search, it was found below the root of the left canine (Figure 3). In case 4, the mental foramen of the contralateral side was missing or obscured by other radiopaque structure we diagnosed as IO. In the affected side, this foramen was smaller and partially blocked by a rim of poorly mineralized material. In case 5, the GOL was seen obscuring the mental foramen in the involved side and surrounding an unerupted, dilacerated left second premolar (Figure 6). Additionally, in cases 6 and 7, a small radiopaque mass was seen blocking the image of the left dental foramen (Figure 2). Right foramen was partially hidden by a radiopaque mass in case 11 (Figure 5) and case 13 showed its right mental foramen partially obscured by the GOL. Interestingly, an additional foramen was seen in the tip of the second premolar (Figure 8). Case 15 had a similar situation; the left mental foramen was almost covered by the lesion. In the case 2, no separation was detected between the lesion and the unerupted supernumerary premolar (Figure 3). Tilting of the related roots was seen in cases 1, 2, 6 and 9 (Figures 2 and 3). Additionally, patient 8 was previously diagnosed with the Prader-Willi syndrome (Figure 8) and an odontogenic keratocyst in the right mandibular premolar-molar zone was previously resected to patient 4 (Figure 9).

## Discussion

This is the first report on three subjects: Diagnosis of GOLs in Latin American population and the presence of osseous and dental developmental alterations. The presence of GOLs in the maxillomandibular area was first reported by Kawai, et al.^17^ (1996), but the association of GOLs and bone and dental developmental alterations have never been published.

The term CO was first applied to “those instances in which sclerotic bone is most often dense and has been formed as a direct result of infection”^3^ and later to “these lesions associated to low grade chronic inflammation of the bone around the apex of a tooth.”^27^ Later, the Glossary of Endodontic Terms of the American Association of Endodontists defined CO (focal sclerosing osteomyelitis) as “a diffuse radiopaque lesion believed to represent a localized bony reaction to a low-grade inflammatory stimulus, usually seen at the apex of a tooth”^2^. Recently, Green, et al.^13^ (2013) proposed a more complex definition: “a diffuse radiopaque lesion believed to represent a localized bony reaction to a low-grade inflammatory stimulus, usually seen at the apex of a tooth (or its extracting site) in which there has been a long-standing pulp pathosis”.

Frequency of CO lesions varied from 0.6%^20^ to 6.9%^3^ and it is more common in female patients.^1^ ^,^ ^3^ ^,^ ^5^ ^,^ ^26^ ^,^ ^27^ Commonly, COs measure from 2 mm^5^ to 6.5 cm^26^ and it was reported that less than 2% were larger than 2 cm^5^. These lesions are more frequently found in mandible and in posterior zone.^7^ ^,^ ^5^ ^,^ ^20^ ^,^ ^26^ ^,^ ^27^ An unexpected frequency of 33% was found in Iranian edentulous patients^3^. Proposed factors to explain the development of CO are numerous: inflamed dental pulp, orthodontic forces, dental eruption, deep dental caries, large dental restorations, dental extraction, teeth with marked malposition or teeth serving as abutments for fixed bridges or partial dentures.^1^ ^,^ ^3^ ^,^ ^5^ ^,^ ^7^ ^,^ ^8^ ^,^ ^20^ ^,^ ^26^ ^,^ ^27^

IO is a radiopaque lesion previously detected in pelvis and long bones mainly.^14^ ^,^ ^19^ It was defined as an asymptomatic, non-expansile, radiopaque or mixed lesion, developing in the tooth bearing area that occurs at any age, it appears in women and men, lacking any relationship with inflammatory, infectious or traumatic phenomena.”^21^ IO is a lesion preferentially found in females and more common in mandible and posterior areas, with preference by the premolar and molar zones.^9^ ^,^ ^11^ ^,^ ^19^ ^,^ ^23^ To date, it is considered as an anatomic variant or a developmental bone lesion.^9^ ^,^ ^10^ ^,^ ^18^

Analyzed GOLs present similarities and differences with those found in IO and CO previously reported series measuring less than 2.5 cm.^1^ ^,^ ^3^ ^,^ ^5^ ^,^ ^7^ ^,^ ^8^ ^,^ ^20^ ^,^ ^26^ ^,^ ^27^ In our sample, IO:CO rate was 2.4:1, but in previously reported studies analyzing smaller cases, COs were more frequently found.^20^ ^,^ ^26^ ^,^ ^27^ As it has been reported, smaller IOs and COs rarely were painful lesions.^1^ ^,^ ^3^ ^,^ ^11^ ^,^ ^18^ ^-^ ^23^ ^,^ ^26^ ^,^ ^27^ Our studied examples were similar in mean age of the patients, gender and mandibular location to those reported in smaller IO and CO cases. Interestingly, our finding on the higher frequency of GOLs in the anterior and premolar zones suggest that differences between GOLs and smaller IOs and COs could exist.

Similarities between our studied GOLs and the gigantic dense bone islands from Kawai, et al.^17^ (1996) study exist. In both studies, more IOs than COs were found. They were more common in females and were mainly located in mandible. In contrast, premolar and molar zones were more frequently affected in the Kawai, et al. report^17^ (1996), and in ours, GOLs were more commonly discovered in the mandibular anterior zone. Additionally, both studies found that round and homogeneous lesions were more frequent. Painful lesions were not found in this study, and in the Kawai, et al.^17^ report, there were 3 cases (14.3%). Excepting the type V GOLs of the Kawai, el al.^17^ (1996) study, all other types were observed in this study because we did not included images outside the tooth-bearing area. Results from the studied cases show that our cases and those reported by Kawai, et al.^17^ (1996), all of them, are the same type of lesions.

There are striking radiological resemblance between GOLs and other radiopaque entities of the maxillofacial bones. GOLs should be differentiated from central exostoses, osteomas, mature central ossifying fibromas, cemento-osseous dysplasias, complex odontomas, gigantiform cementomas, osteopoikilosis (spotted bone disease), osteosarcoma, metastatic carcinomas, osteomyelitis and medullary bone infarct, among others.^14^ ^-^ ^17^ ^,^ ^20^ This is a very important issue since some of them are malignant entities and the correct diagnosis will prevent inadequate treatment.

An unusual and interesting observation was done during the review and scrutinization of this radiographic material. It was noted that numerous developmental alterations of dental origin were present. The most common dental alterations were microdontia, hypodontia, enamel pearls, pulp stones and macrodontia. Additionally, one patient was diagnosed with the Prader-Willi syndrome and other was previously operated of an odontogenic keratocyst. Also, both mental and incisive foramina showed various morphological and structural alterations. Together, all these findings on developmental alterations suggest the possibility of a genetic background.

## Conclusions

It is possible that the abnormal bone deposit distinctive of both studied GOLs could be related with excessive bone activity producing accumulation. For development of CO, two mechanisms working together could be related with its excessive osseous accumulation. The first is modification of the normal turnover producing increase of osseous deposition. The second will prevent the normal bone resorption, arresting the osseous breakdown process.

Note that, as any retrospective study, ours only estimates the relative incidence of this entity in a defined period.
